# Differential Effects of a Left Frontal Glioma on the Cortical Thickness and Complexity of Both Hemispheres

**DOI:** 10.1093/texcom/tgaa027

**Published:** 2020-06-27

**Authors:** Ryuta Kinno, Yoshihiro Muragaki, Takashi Maruyama, Manabu Tamura, Kyohei Tanaka, Kenjiro Ono, Kuniyoshi L Sakai

**Affiliations:** Department of Basic Science, Graduate School of Arts and Sciences, The University of Tokyo, Tokyo, 153-8902, Japan; Division of Neurology, Department of Internal Medicine, Showa University Northern Yokohama Hospital, Yokohama, 224-8503, Japan; Department of Neurosurgery, Tokyo Women's Medical University, Tokyo, 162-8666, Japan; Department of Neurosurgery, Tokyo Women's Medical University, Tokyo, 162-8666, Japan; Department of Neurosurgery, Tokyo Women's Medical University, Tokyo, 162-8666, Japan; Department of Basic Science, Graduate School of Arts and Sciences, The University of Tokyo, Tokyo, 153-8902, Japan; Division of Neurology, Department of Medicine, Showa University School of Medicine, Tokyo, 142-8666, Japan; Department of Basic Science, Graduate School of Arts and Sciences, The University of Tokyo, Tokyo, 153-8902, Japan

**Keywords:** cortical structural change, cortical thickness, fractal dimension, glioma, surface-based morphometry

## Abstract

Glioma is a type of brain tumor that infiltrates and compresses the brain as it grows. Focal gliomas affect functional connectivity both in the local region of the lesion and the global network of the brain. Any anatomical changes associated with a glioma should thus be clarified. We examined the cortical structures of 15 patients with a glioma in the left lateral frontal cortex and compared them with those of 15 healthy controls by surface-based morphometry. Two regional parameters were measured with 3D-MRI: the cortical thickness (CT) and cortical fractal dimension (FD). The FD serves as an index of the topological complexity of a local cortical surface. Our comparative analyses of these parameters revealed that the left frontal gliomas had global effects on the cortical structures of both hemispheres. The structural changes in the *right* hemisphere were mainly characterized by a decrease in CT and mild concomitant decrease in FD, whereas those in the peripheral regions of the glioma (*left* hemisphere) were mainly characterized by a decrease in FD with relative preservation of CT. These differences were found irrespective of tumor volume, location, or grade. These results elucidate the structural effects of gliomas, which extend to the distant contralateral regions.

## Introduction

Diffuse gliomas, the most prevalent primary malignant brain tumors, have been classified into grades II–IV, with gliomas of grades II and III being slow-growing and generally less aggressive (Suzuki et al. 2015; Motomura et al. 2019). Slow-growing tumors have been suggested to be the most epileptogenic (van Breemen et al. 2007). Gliomas have generally been considered to cause hyperexcitability throughout the entire brain, leading to epileptic seizures (de Groot et al. 2012), whereas a magnetoencephalography study of gliomas in the resting state has shown that these lesions disrupt functional connectivity across distant regions in both hemispheres (Bartolomei et al. 2006). We have previously reported that agrammatic patients with a left frontal glioma showed global changes in functional connectivity among syntax-related networks (Kinno et al. 2014). Our subsequent studies further indicated that the connections between the left fronto-parietal regions, as well as between the left triangular and orbital parts of the left inferior frontal gyrus (F3t and F3O), are critical for preserving the syntactic abilities of those patients (Kinno et al. 2015). In spite of these functional changes, which should shed new light on neurological symptoms, it remains unclear how such abnormalities are influenced by any anatomical changes in the *preoperative* period, which may be global as well; note that anatomical deformation in the *postoperative* period is usually too large to examine in a systematic manner (see Fig. 2 of Saito et al. 2016).

Neuroimaging—including neuroimaging using voxel-based morphometry (VBM) (Ashburner and Friston 2000) and cortical pattern matching (Thompson et al. 1998)—has been a powerful method for providing neurological insights related to anatomical changes. Recent VBM studies have shown that patients with a glioma in the left hemisphere exhibited greater gray matter (GM) volume in distant regions of the right insular cortex (Almairac et al. 2018) or cerebellum (Zhang et al. 2018). Whereas VBM has been used to establish a pattern of structural changes (Bora et al. 2011), such changes probably reflect a mixture of effects, including both genuinely pathogenetic mechanisms and transitional phases. Such changes could variously reflect the effects of disease progression, medication, or even the short-term occurrence of a psychotic episode (Nenadic et al. 2014). Recent developments in surface-based morphometry (SBM) have enabled the measurement of both cortical thickness (CT) and the cortical complexity of folding and gyrification (Luders et al. 2006b; Gutman et al. 2009; Lui et al. 2010). Because such cortical complexity seems to be stable throughout the life span after puberty (Armstrong et al. 1995), any abnormal complexity is likely to be detected if proper methods are employed. One promising approach is to apply the fractal dimension (FD), that is, the estimate of the topological complexity of an object in general, to the measurement of cortical complexity. The cortical FD could become a potential marker to indicate the degree of brain damage in patients with psychiatric or neurological symptoms, because of its sensitivity in detecting brain changes (Di Ieva et al. 2015). Considering the mass effects and infiltrative nature of a glioma, we suspect that the glioma-induced cortical structural changes would affect both the CT and FD.

In the present study, we aimed to clarify *how* the cortical structures are globally changed for patients with a glioma in the left lateral frontal cortex. For this purpose, we compared their CT and FD with those of healthy controls. It is possible that the cortical structural changes in such patients reflect not only infiltration but also compression of the brain, both of which may be detected by the conventional MRI images. We therefore preoperatively examined the cortical structure of the entire brain, including the contralateral regions that showed functional connectivity changes, as reported in our previous studies (Kinno et al. 2014, 2015). The exact identification and surgical removal of a glioma in the left frontal cortex requires a most careful preoperative examination, because it is crucial to preserve the patient’s language function (Saito et al. 2016). Our present findings would also provide fundamental insights on how to understand the global architecture of the cerebral cortex (e.g., lateralization) with respect to the language area.

## Materials and Methods

### Participants

We examined 15 patients (hereafter, “patient group”) who were native Japanese speakers newly diagnosed as having a glioma in the left frontal cortex (9 males and 6 females, age 24–60 years; median age: 39 years) (Table 1). The patients were preoperatively examined with structural/functional MRI scans at the University of Tokyo, Komaba (the functional data are currently being prepared for publication), and they underwent surgery to remove the glioma at the Department of Neurosurgery, Tokyo Women’s Medical University between 2014 and 2016. All 15 patients met each of the following inclusion criteria: (i) right-handedness, (ii) no deficits in verbal/written communication or other cognitive abilities reported by the patients or physicians, (iii) no history of neurological or psychiatric disorders other than glioma and seizures, (iv) free from seizures with or without antiepileptic drugs, (v) no history of brain radiotherapy, (vi) no medical problems related to MRI acquisition, (vii) no intercranial abnormalities other than glioma, and (viii) histologically proven grade II or grade III diffuse astrocytic and oligodendroglial tumors (i.e., astrocytoma, oligodendroglioma, or oligoastrocytoma) according to the 2007 WHO Classification of Tumors of the Central Nervous System (Louis et al. 2007). The laterality quotient of handedness was determined by the Edinburgh Handedness Inventory (Oldfield 1971). The glioma of each patient was located mostly in the left lateral frontal cortex.

**Table 1:** Patient characteristics

Patient number	LQ	L. F3op	L. F3t	L. F3O	L. LPMC	Tumor location			Tumor volume	Tumor type	Tumor grade
						*x*	*y*	*z*			
1	100		+	+		-30	30	-10	3529	OL	II
2	100	+				-32	48	12	4927	DA	II
3	100	+	+			-40	10	4	6553	AA	III
4	80	+	+	+		-28	38	4	21 539	AO	III
5	100	+	+	+		-26	16	-10	19 380	AOA	III
6	60	+	+	+	+	-22	36	10	28 714	AOA	III
7	100	+	+		+	-36	4	12	38 371	AOA	III
8	100	+	+		+	-42	-4	6	35 804	AA	III
9	78	+	+		+	-44	12	12	15 505	OL	II
10	100	+			+	-47	2	8	5902	DA	II
11	100	+			+	-26	-9	45	33 125	AOA	III
12	65				+	-32	12	48	8228	OL	II
13	100				+	-28	0	52	5189	AA	III
14	67				+	-38	2	42	1413	OL	II
15	100				+	-15	15	47	21 508	OL	II
Median	100								11 867		

The laterality quotient of handedness was determined by the Edinburgh Handedness Inventory (Oldfield 1971). MR images were normalized with SPM12 to determine the tumor location, as well as the tumor volume (mm^3^) of a tumor including the gray matter and white matter. The determination of tumor types and grades (II or III, with III being more severe) was based on the 2007 WHO Classification of Tumors of the Central Nervous System (Louis et al. 2007). Tumor locations were determined based on the previous study (Glasser et al. 2016). The “+” sign indicates the presence of a glioma in each cluster. MNI coordinates of each tumor location (the center of geometry) are shown. AA = anaplastic astrocytoma (grade III); AO = anaplastic oligodendroglioma (grade III); AOA = anaplastic oligoastrocytoma (grade III); DA = diffuse astrocytoma (grade II); F = female; F3O = orbital part of the inferior frontal gyrus; F3op = opercular part of the inferior frontal gyrus; F3t = triangular part of the inferior frontal gyrus; L. = left; LQ = laterality quotient; LPMC = left lateral premotor cortex; M = male; OA = oligoastrocytoma (grade II); OL = oligodendroglioma (grade II).

We also recruited 15 age- and gender-matched healthy participants (hereafter, “control group”) for our structural MRI experiments (12 males and 3 females, age 23–55 years; median age: 28 years). Because the ages were not normally distributed (Shapiro-Wilk test, *P* = 0.010), we used nonparametric tests to confirm the absence of age differences (Mann-Whitney’s *U* test: males, *P* = 0.14; females, *P* = 0.070) and gender differences (Fisher’s exact test: *P* = 0.21) between the 2 groups. Written informed consent was obtained from each participant after the nature and possible consequences of the studies were explained. Approval for the experiments was obtained from the institutional review boards of the University of Tokyo, Komaba, and Tokyo Women’s Medical University.

### MRI Data Acquisition

The MRI scans were conducted on a 3.0 T system (GE Signa HDxt 3.0 T; GE Healthcare, Milwaukee, WI, USA). The high-resolution T1-weighted images of the whole brain (136 axial slices, 1.0 × 1.0 × 1.0 mm^3^) were acquired from all participants with a three-dimensional fast spoiled gradient recalled acquisition in the steady-state (3D FSPGR) sequence (repetition time = 8.4 ms, echo time = 2.6 ms, flip angle = 25°, field of view = 256 × 256 mm^2^).

### MRI Data Processing

The structural MRI data preprocessing was performed in a standard manner using the CAT12 Toolbox (http://dbm.neuro.uni-jena.de/cat/) included in the SPM12 statistical parametric mapping software (Wellcome Trust Centre for Neuroimaging, http://www.fil.ion.ucl.ac.uk/spm/) (Friston et al. 1995) and implemented on MATLAB software (MathWorks, Natick, MA, USA). Preprocessing was performed by the CAT12 Toolbox under the default settings, except that the East Asian brain template was used for affine registration. All of the T1-weighted anatomical images were manually reoriented to place the anterior commissure at the origin of the 3D Montreal Neurological Institute (MNI) space. The images were then segmented into GM, white matter (WM), and cerebrospinal fluid (CSF) (Ashburner and Friston 2005). Next, the images were normalized to the MNI space by using a diffeomorphic nonlinear registration algorithm of the diffeomorphic anatomical registration through exponentiated Lie algebra (DARTEL) toolbox (Ashburner 2007). The DARTEL algorithm has been used in previous VBM studies for patients with a glioma (Almairac et al. 2018; Zhang et al. 2018; Yuan et al. 2020). The final resulting voxel size was 1.5 × 1.5 × 1.5 mm^3^. All resultant images were visually inspected for the absence of artifacts and passed the quality control and homogeneity control algorithms implemented in the CAT12 Toolbox. The normalized T1-weighted anatomical images were then used to determine the tumor location for each patient.

### Lesion Analyses

Each glioma was identified on normalized T1-weighted structural images by experienced neurologists and neurosurgeons, and the glioma boundary was semiautomatically determined using the 3D Fill tool in the MRIcron software package (http://www.mccauslandcenter.sc.edu/mricro/mricron/), which generated a contiguous cluster of voxels defined by the intensity of the glioma itself. The boundaries of each lesion, including brain edemas and abnormalities of perfusion, were confirmed with the T2-weighted MR images taken at the Department of Neurosurgery. The absence of skip lesions distant from the glioma was confirmed with ^11^C-methionine, [^18^F] fluorodeoxyglucose, and ^11^C-choline PET data (resolution = 4.8 × 4.8 × 4.25 mm^3^) taken at the Chubu Medical Center for Prolonged Traumatic Brain Dysfunction (Minokamo City, Gifu, Japan). Lesion overlap maps were computed with the MRIcron software, then converted to the standard surface template, and visualized with the CAT12 Toolbox. The center of geometry for each tumor was calculated with MRIcron software. We estimated the volume and center of geometry for each tumor on the normalized T1-weighted image. No visible midline shift was present on the normalized MR image of any of the patients, as confirmed on the axial slices at the level of the midbrain (*z* = −10) and the body of the corpus callosum (*z* = 35).

### Cortical Thickness and Central Surface Estimation

The cortical surface extraction from the T1-weighted MR images, as well as the estimation of CT and FD, were made by using the CAT12 Toolbox with the projection-based thickness (PBT) under the default settings (Dahnke et al. 2013). For the cortical surface extraction from each hemisphere, we created the “central surface” (CS), which was positioned at an equal distance between the outer surface (i.e., the boundary between the CSF and GM) and the inner surface (i.e., the boundary between the GM and WM). More specifically, the procedures included the following 4 steps: (1) tissue segmentation into GM, WM, and CSF; (2) separation of the cortex into the left hemisphere, right hemisphere, and cerebellum, using the segmented images; (3) creation of the masked version of segmented images including ventricular and subcortical regions with an interpolation to 0.5 × 0.5 × 0.5 mm^3^; and (4) creation of the CS with CT information by PBT. For the last procedure (4), the distance from the inner surface was first estimated for each GM voxel, by using a voxel-based distance method (see the *Distance measure* section of the abovementioned paper) (Dahnke et al. 2013). The distance at a GM voxel on the outer surface thus corresponded to the CT value. We then obtained the voxels constituting the CS, whose distances from the inner surface were one-half of the CT values.

For an interparticipant analysis, a spherical map of a cortical surface is usually necessary to reparametrize the surface mesh into a common coordinate system. A topology correction based on the spherical harmonics was used to correct the topology of the cortical surface generated with the PBT (Yotter et al. 2011a). A fast algorithm was used to reduce the area distortion of the input spherical map, leading to an improved reparameterization of the surface mesh (Yotter et al. 2011c). These procedures were performed with the CAT12 Toolbox. For the spherical registration, an adapted two-dimensional diffeomorphic DARTEL algorithm (Ashburner 2007) was then applied to the surface. Finally, all data were smoothed with a Gaussian kernel of 20 mm full width at half maximum (FWHM).

### FD Estimation

We calculated the FD values by using spherical harmonic (SPH) expansions (Yotter et al. 2010, 2011b; Nenadic et al. 2014). The computation of the FD for a brain surface mesh was performed with the CAT12 Toolbox and involved the following 3 steps: (1) generation of a CS mesh for each hemisphere under the default settings (see Cortical Thickness and Central Surface Estimation); (2) extraction of the spherical harmonic coefficients of the CS up to a maximum bandwidth of 1024 (for details on the SPH analysis, see the abovementioned paper) (Yotter et al. 2011b); and (3) calculation of FD values by finding the slope of a plot regressing log(*area*) versus log(*maximum bandwidth*). In a general multifractal analysis, FD values are computed by finding the slope of a plot regressing log(*area*) versus log(*dimension*) over a certain range of scales, where the area is the total area of an object and the dimension is the scale of measurement, which is varied by either subsampling the object or reducing the degree of the shape representation. For SPH reconstructions of the cortical surface, the plot was modified to use the maximum bandwidth of the reconstruction (i.e., a measure of the bandwidth of frequencies used to reconstruct the surface shape) (see the Appendix of the abovementioned paper) (Yotter et al. 2011b).

The FD maps from each participant were also reparameterized into a common coordinate system. This was accomplished by using the registered spherical meshes (*rh.sphere.reg* and *lh.sphere.reg*), followed by mapping to the standard template (the surface mesh of *fsaverage* [average subject]). The registered spherical mesh and standard template were included in the CAT12 Toolbox. Finally, all data were smoothed with a Gaussian kernel of 20 mm FWHM.

### Statistical Analyses

The statistical analyses were performed using the general linear model approach implemented in SPM12. For the SBM analyses, two-sample *t*-tests were used to compare the control and patient groups, whereas paired *t*-tests were used to compare data within each group. Age and gender are known to affect cortical structures (Salat et al. 2004; Luders et al. 2006a), and we thus included age and gender as nuisance factors in the design matrix of SBM analyses. For the presentation of statistical maps, we used threshold-free cluster enhancement (TFCE) with 10 000 permutations for a combined analysis of the height and size of the effects (Smith and Nichols 2009), applying a false discovery rate (FDR) corrected threshold of *P* < 0.05. Using the CAT12 Toolbox, the anatomical location of a glioma or cluster was determined with reference to the multimodal analyses of magnetic resonance images from the Human Connectome Project (HCP) (Glasser et al. 2016).

## Results

### The Glioma Location

**Figure 1:**
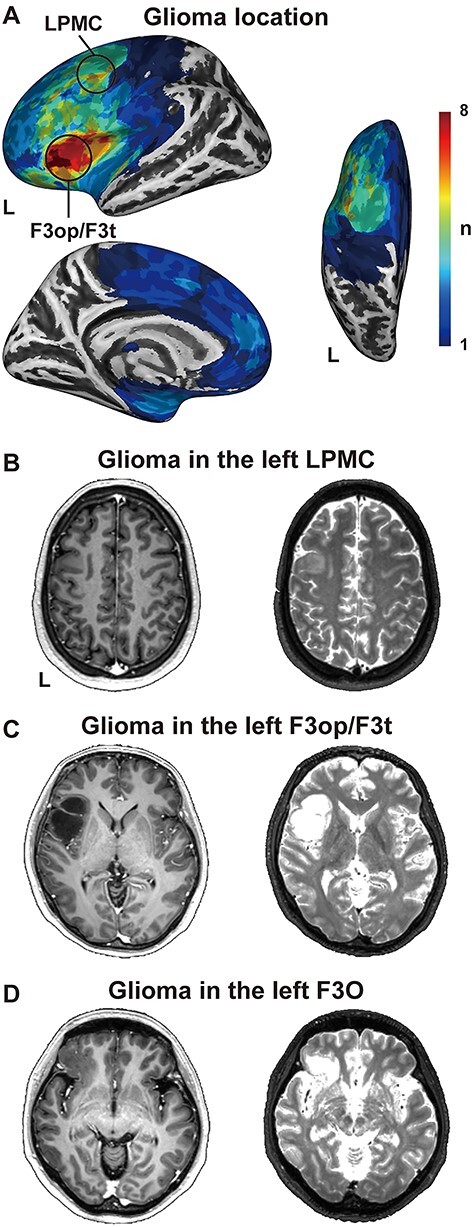
A glioma in the left frontal cortex. (*A*) Lesion overlap maps for the patient group. For the left (*L*) hemisphere of an inflated standard brain, the lateral (left top), medial (left bottom), and dorsal (right) views are shown. The color bar denotes the number of patients. Note the major overlap of lesions in the opercular and triangular parts of the left inferior frontal gyrus (F3op/F3t) and in the left lateral premotor cortex (LPMC). (*B–D*) Representative brain MRI images of patients. The T1-weighted (left) and T2-weighted images (right) of individual patients with a glioma in the left LPMC (*B*), left F3op/F3t (*C*), and left F3O (*D*) are shown on the individual axial slices. Note the absence of intracranial abnormalities in the right hemispheres.

The glioma locations in the left frontal cortex are shown in Figure 1 and Table 1. For the 15 patients we tested, the gliomas were located in 5 major cortical regions: the (i) left lateral premotor cortex (LPMC), (ii) left dorsolateral prefrontal cortex, (iii) left inferior frontal gyrus, (iv) left orbital and polar frontal cortex, and (v) left frontal opercular and insular cortex. Among these clusters, the maximum lesion overlap was located in the opercular, triangular, and orbital parts of the left inferior frontal gyrus (F3op/F3t/F3O; Brodmann’s areas [BAs] 44, 45, and 47; see Table 1 and Fig. 1*C,D*), and it was extended into the left superior insula. The second largest lesion overlap was in the left LPMC (BAs 6 and 8; see Fig. 1*B*). Compared with these lateral regions, the left medial frontal regions minimally included gliomas. Therefore, any effects observed below were due to the presence of a glioma in the left lateral frontal cortex.

**Figure 2:**
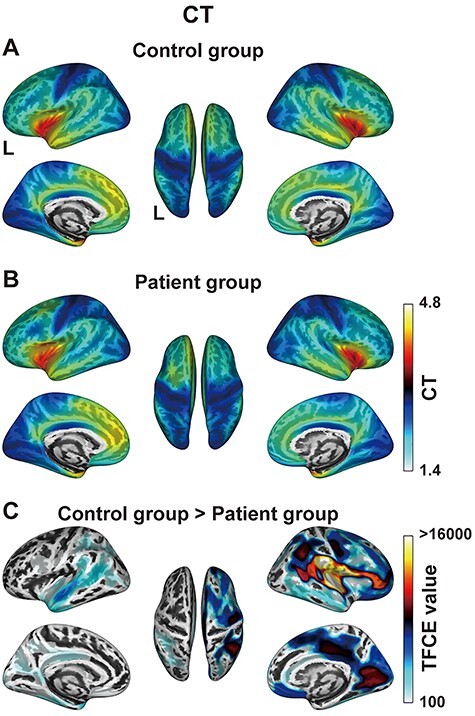
Surface-based morphometry analyses of the CT. (*A, B*) The CT maps averaged for the control group (*A*) and patient group (*B*), which were projected onto the inflated standard brain. Lateral (left top and right top), medial (left bottom and right bottom), and dorsal (center) views are shown (the same configuration for subsequent panels). The color bar denotes the mean CT values (in mm) for each group. (*C*) Brain regions with significantly decreased CT for the patient group, estimated with TFCE values (FDR-corrected *P* < 0.05). The color bar denotes the TFCE values, where the minimum TFCE value of this comparison was 117. Note the significant decrease in CT in the right hemisphere for the patient group. There was no region with significantly increased CT for the patient group. CT = cortical thickness.

**Figure 3:**
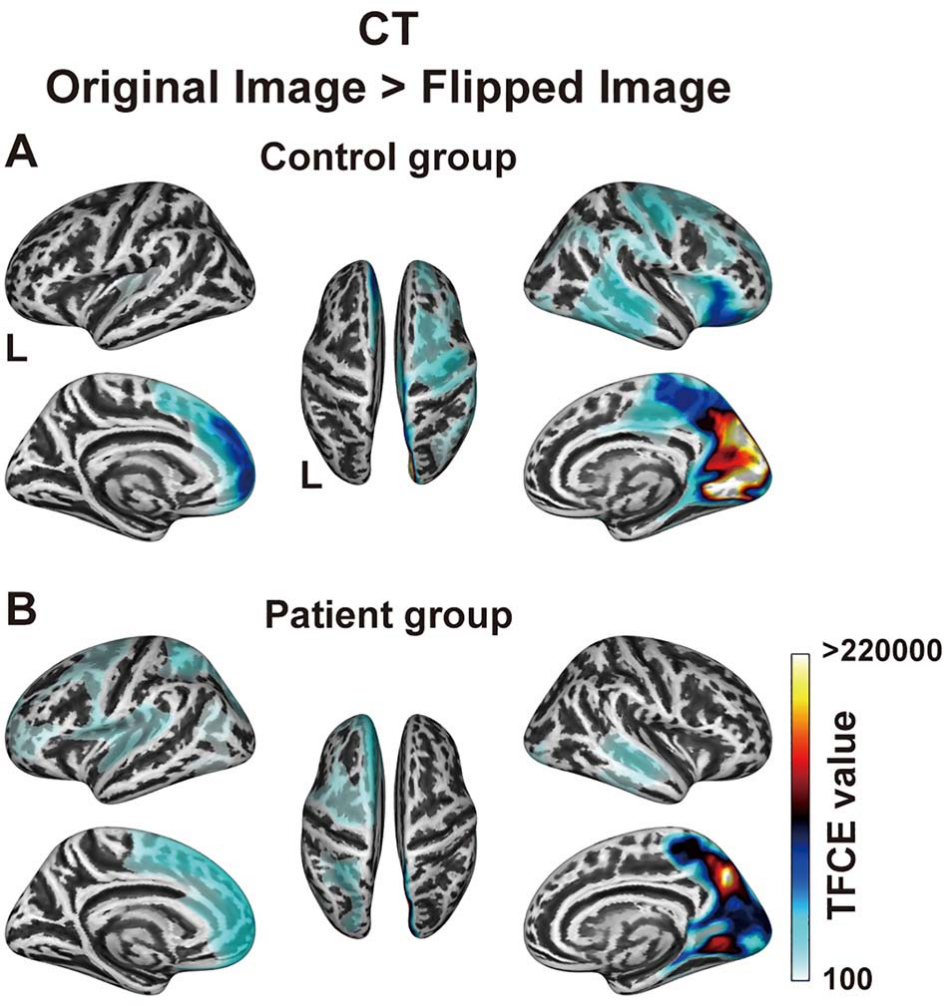
Hemispheric asymmetry of the CT. (*A, B*) Brain regions identified by the comparison between the original and flipped images (FDR-corrected *P* < 0.05) for the control group (*A*) and patient group (*B*). The minimum TFCE values of the comparison for the control and patient groups were 1190 and 1776, respectively. Note the significantly rightward asymmetry in the lateral frontal cortex for the control group, which disappeared almost completely for the patient group.

**Figure 4:**
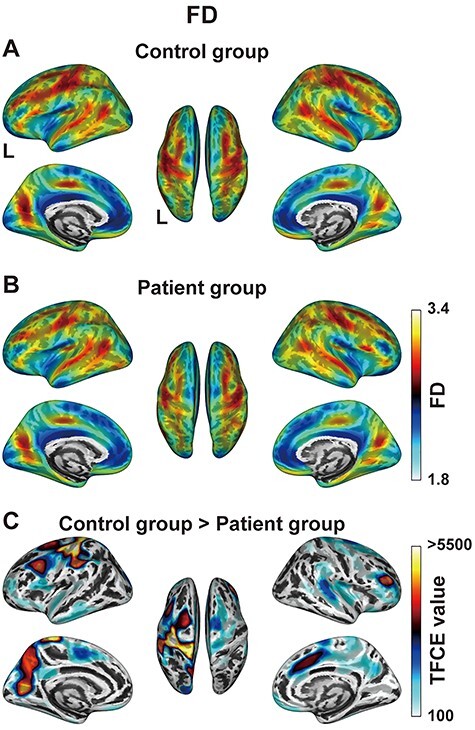
Surface-based morphometry analyses of the FD. (*A, B*) The FD maps averaged for the control group (*A*) and patient group (*B*). The color bar denotes the mean FD values for each group. (*C*) Brain regions with the significantly decreased FD for the patient group (FDR-corrected *P* < 0.05). The color bar denotes the TFCE values, where the minimum TFCE value of this comparison was 103. Note the significant decrease in FD in the left frontal cortex for the patient group, corresponding to the regions with a glioma. There was no region with significantly increased FD for the patient group. FD = fractal dimension.

**Figure 5:**
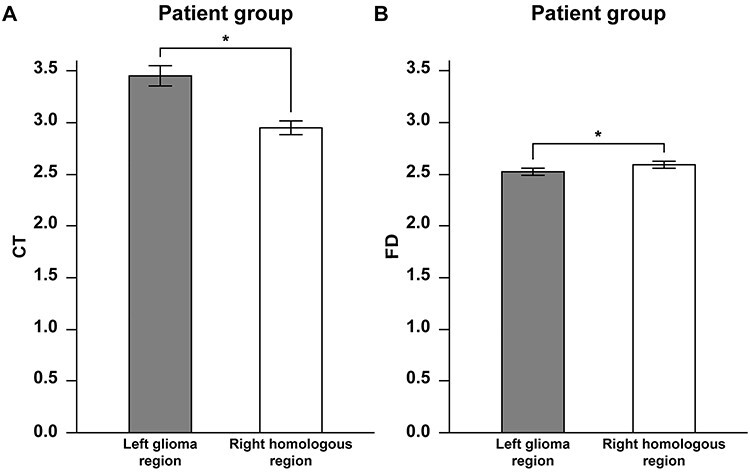
Comparisons between the glioma region and its right homolog. (*A*) Histograms of the CT obtained from individual patients. Error bars indicate the standard error of the mean for the participants. *P < 0.05 (Wilcoxon signed rank sum test). (*B*) Histograms of the FD obtained from individual patients. Note the contrasting results of the decreased CT in the right homologous region and of the decreased FD in the glioma region.

**Figure 6:**
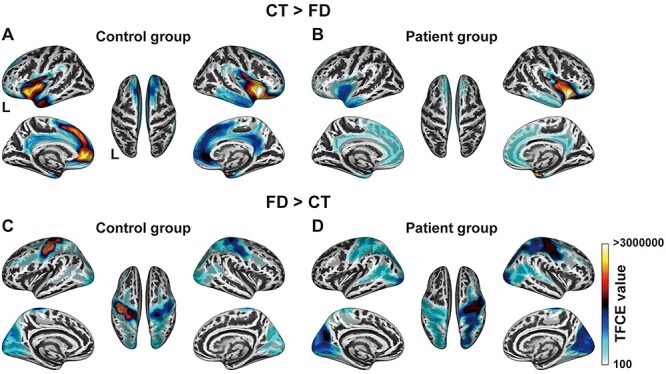
Direct comparisons between the CT and FD. (*A, B*) Significant regions identified by the paired *t*-test of the relative comparison of CT > FD (FDR-corrected *P* < 0.05) for the control group (*A*) and patient group (*B*). The minimum TFCE values of the comparison for the control and patient groups were 4406 and 1634, respectively. (*C, D*) Significant regions identified by the paired *t*-test of FD > CT (FDR-corrected *P* < 0.05) for the control group (*C*) and patient group (*D*). The minimum TFCE values for the control and patient groups were 3924 and 5215, respectively. Note the significant drop of CT > FD in the left inferior frontal gyrus and left medial frontal cortex for the patient group.

We examined whether the tumor location was related with any of the following factors: the tumor volume, the laterality quotient of handedness, age, and gender. According to Spearman’s rank correlation tests, the tumor location (i.e., the MNI coordinates for the center of geometry) was not significantly affected by the tumor volume (*x*-, *y*-, and *z*-axis: all, *P* > 0.40), laterality quotient (all, *P* > 0.34), or age (all, *P* > 0.21). Mann–Whitney’s *U* tests showed that gender did not significantly influence the tumor location (all, *P* > 0.95). These results suggest that the subsequent effects revealed by SBM analyses (Figs 2–6) were due to the presence of a randomly localized glioma in the left frontal cortex.

### Decreased CT in the Right Hemisphere of the Patient Group

We first examined the CT maps averaged for the control and patient groups (Fig. 2*A,B*). Each group had higher CT values in the bilateral insular and frontal opercular cortices, while a lower CT was observed in the bilateral parietal and occipital cortices. When compared between these 2 maps, the CT in the *right frontal* regions of the patient group appeared to be lower than that of the control group (the dorsal views of Fig. 2*A,B*), which was statistically verified (Fig. 2*C*). Moreover, the patient group had the significantly decreased CT in almost all regions except for the left F3op/F3t and left LPMC with gliomas (Fig. 1*A*). Among these regions, the most prominent decrease in CT was observed in the right perisylvian cortex, which extended from the frontal opercular and insular cortex to the entire right hemisphere. These results indicate that a glioma causes a CT decrease especially in the contralateral right hemisphere. In contrast, there was no region with significantly increased CT for the patient group.

To clarify the lateralization of the CT, we compared the original images with flipped ones for each group (Fig. 3). The control group showed significantly rightward asymmetry in the lateral frontal cortex, medial parietal cortex, and medial occipital cortex (Fig. 3*A*). In contrast, the rightward asymmetry of the lateral frontal cortex was almost completely absent for the patient group (Fig. 3*B*), whereas the lateralization in the medial parietal and occipital cortices, together with the leftward asymmetry of the medial frontal cortex, were well preserved. Moreover, the patient group showed the leftward asymmetry of the lateral frontal and parietal cortices. These results confirmed the prominent effect of a glioma on the CT in the right lateral frontal cortex.

### Decreased FD in the Left Frontal Cortex of Patients

Next, we examined the FD map averaged within each of the control and patient groups (Fig. 4*A,B*). Each group had higher FD values in the bilateral superior frontal and parietal cortices, while lower FD was observed in the bilateral insular and frontal opercular cortices. The latter observation was consistent with the *higher* CT in the same regions; the thicker the cortex is, the less its complexity becomes. When compared between these 2 maps, the FD in the *left frontal* regions of the patient group appeared to be lower than that of the control group (the dorsal views of Fig. 4*A,B*), which was statistically verified (Fig. 4*C*). Indeed, the patient group had the significantly decreased FD in the left LPMC and F3op/F3t, corresponding to the region with a glioma. In the left lateral premotor and dorsolateral prefrontal cortices, a prominent decrease in FD was observed in the peripheral regions of gliomas. In the right hemisphere, a decrease in FD was observed in the dorsolateral prefrontal and perisylvian cortices. It should thus be noted that the CT and FD values were not always complementary or parallel; rather, these values provided additional information. In contrast, there was no region with significantly increased FD for the patient group. For each group, we also examined the lateralization of the FD, but there was no statistically significant trend.

### Comparisons Between the Glioma Region and Its Right Homolog

The hemispheric differences observed for the patient group (Figs 2*C* and 4*C*) suggest the distant effects of a glioma that extends from the left frontal cortex to the right hemisphere. To clarify such effects for individual patients, we obtained the CT and FD not only from the region within a glioma but from the right homologous region. By comparing these 2 regions in the patient group, we observed that the CT in the right homolog was significantly lower than that of the glioma region (Paired *t*-test, *t* = 5.3, *P* = 0.0001; Fig. 5*A*). In contrast, the FD in the glioma region was significantly lower than that of its right homolog (*t* = 2.7, *P* = 0.018; Fig. 5*B*). We further examined whether the difference in CT between the glioma region and its right homolog was related with any of the following factors: tumor volume, location (the center of geometry), and grade. Neither tumor volume (Pearson’s correlation test, *r* = −0.029, *P* = 0.93) nor location (*x*-axis: *r* = 0.18, *P* = 0.52; *y*-axis: *r* = −0.19, *P* = 0.50; *z*-axis: *r* = 0.25, *P* = 0.37) was correlated with the CT difference between the two hemispheres. Moreover, there was no significant CT difference between grades II and III (*t*-test, *t* = 0.42, *P* = 0.68). Regarding the FD difference, there was no significant correlation with tumor volume (*r* = −0.22, *P* = 0.43), location (*x*-axis: *r* = 0.18, *P* = 0.52; *y*-axis: *r* = −0.19, *P* = 0.50; *z*-axis: *r* = 0.25, *P* = 0.37), or grade (*t* = 1.6, *P* = 0.14). Therefore, we conclude that the presence of a glioma in the left frontal cortex caused the lower CT in the *right* frontal regions (Fig. 2), as well as the lower FD in the *left* frontal regions (Fig. 4), indicating that the glioma had differential effects on the 2 hemispheres.

### Direct Comparisons Between the CT and FD

A decreased CT represents the thinning of gray matter, whereas a decreased FD indicates a reduction of cortical complexity. Because cortical structural changes due to a glioma are generally characterized by the infiltration and compression to cortical regions, the compression of surrounding tissues may result in the decrease in CT, while the infiltration may cause the decrease in FD. On the grounds that CT and FD influence each other (King et al. 2009), we tried to distinguish the relative contribution of these 2 effects by directly comparing the values of CT and FD. Because the 2 metrics are independent with different scales, we took the simple difference between the 2 values and then used a map of these differences in the control group as a reference (Fig. 6*A,C*). For the control group, the bilateral inferior frontal and medial frontal cortices showed significantly higher CT than FD values (Fig. 6*A*). In contrast, for the patient group, a significant drop of the CT > FD difference was observed in the *left* inferior frontal gyrus and medial frontal cortex (Fig. 6*B*), which was consistent with the glioma location (see Fig. 1). In contrast, the difference in the right inferior frontal gyrus was well preserved.

Finally, we examined the FD > CT difference, and found significantly higher FD than CT values for the control group in the bilateral premotor and occipital cortices, especially in the left premotor cortex (Fig. 6*C*). For the patient group, a significant drop of the FD > CT difference was observed in the *left* lateral premotor cortex (Fig. 6*D*), in parallel with a slight increase of the difference in the right lateral premotor and bilateral occipital cortices. In sum, the preserved CT > FD difference for the patient group in the right hemisphere, as well as the preserved or slightly increased FD > CT differences, suggested that the structural changes in the *right* hemisphere were mainly characterized by the decrease in CT with the mild concomitant decrease in FD. On the other hand, the significant drop in the FD > CT difference in the peripheral regions of gliomas suggested that the structural changes in the peripheral regions of the glioma were mainly characterized by the decrease in FD with the relative preservation in CT. These results provide further evidence of hemispheric differences in the cortical changes of the patient group.

## Discussion

Our comparative analyses of the CT and FD data revealed global effects of a left frontal glioma on the cortical structures of both hemispheres, as summarized and then discussed in detail below. The CT in the *right frontal* regions of the patient group was significantly lower than that of the control group, with the most prominent decrease in CT being observed in the right perisylvian cortex (Fig. 2*C*). Moreover, the rightward asymmetry of the CT in the *lateral* frontal cortex for the control group (Fig. 3*A*) was almost completely absent for the patient group (Fig. 3*B*). On the other hand, the FD in the *left frontal* regions of the patient group was significantly lower than that of the control group (Fig. 4*C*), with the most prominent decreases of the FD being observed in the peripheral regions of gliomas and in the right dorsolateral prefrontal and perisylvian cortices (Fig. 4*C*). Indeed, the comparisons between the glioma region and its right homolog revealed that the CT of the right homolog was significantly lower than that of the glioma region, while the FD of the glioma region was significantly lower than that of its right homolog (Fig. 5). These hemispheric differences were found irrespective of tumor volume, location, or grade. Furthermore, the direct comparisons between the CT and FD showed that the CT > FD difference in the left frontal cortex was reduced for the patient group (Fig. 6*A,B*), while the FD > CT difference in the right hemisphere was preserved or increased (Fig. 6*C,D*). These results elucidate the structural effects of gliomas, which extend to the distant contralateral regions. We propose that comparative analyses of CT and FD data could be useful for identifying all cortical regions affected by a glioma.

The cortical structural changes in the hemisphere contralateral to a glioma were mainly characterized by the decreased CT (Fig. 2*C*). Decreased CT generally reflects cortical atrophy due to aging (Salat et al. 2004), which is often associated with cognitive decline (Kinno et al. 2019). However, the effects of age difference were controlled as nuisance factors in our SBM analysis, and the patients showed no apparent cognitive decline. Moreover, the effects of seizures (Bernhardt et al. 2010) and radiation therapy (Karunamuni et al. 2016), both of which have been known to cause cortical thinning, were excluded from the present study. It is known that the mass effects of a glioma include compression of the surrounding tissue, which leads to tangential stretching and thereby thinning of the cortex (Hogea et al. 2007). A recent diffusion tensor imaging (DTI) study on glioma patients suggested that reduced fractional anisotropy in the *contralateral* hemisphere may be due to the compression and cortical thinning caused by a tumor-infiltrated edema (Miller et al. 2012). A tumor-infiltrated edema involves glial alterations in vital brain tissue, that is, astrocytic swelling, microglial accumulation, and microglial activation (Engelhorn et al. 2009). Although there was no noticeable deformation of the midline of the brain in any of our patients (see Fig. 1*B–D*), and tumor-infiltrated edema could not be detected on the conventional T1- or T2-weighted MR images of the contralateral hemisphere, it is possible that the decreased CT in the right hemisphere was due to compression from the tumor-infiltrated edema in the left hemisphere.

Regarding the lateralization of the CT, a previous study reported rightward asymmetry in the lateral frontal cortex, medial parietal cortex, and occipital cortex in the healthy brain (Zhou et al. 2013), which is consistent with our previous report (Nauchi and Sakai 2009) and present findings (Fig. 3*A*). The absence of such rightward asymmetry in the patient group (Fig. 3*B*), as well as the decreased CT in the right homologous regions (Fig. 5*A*), is consistent with the mass effect of a glioma on the contralateral CT. Moreover, the slight *increase* of the FD > CT difference in the right hemisphere (Fig. 6*C,D*) can be explained by the decreased CT in the right hemisphere of the patient group. On the other hand, the decreased FD in the right hemisphere of the patient group (Fig. 4*C*) may be due to a decrease in CT concomitant with the preservation of cortical gyrification, as confirmed by a previous study (King et al. 2009). It is possible that the mass effects of a glioma might cause the cortical thinning in the ipsilateral hemisphere as well, but we did not observe such an effect in the left hemisphere of our patients (Fig. 2*C*). On the contrary, the left hemisphere showed an increased leftward asymmetry near the IFG for the patient group (Fig. 3*B*), which may have been due to astrocytic swelling in the GM edema with less gyrification around the glioma regions. This possibility is consistent with the concomitant decrease in FD (Fig. 4*C*). Moreover, the inherent rightward asymmetry of the CT in the frontotemporal regions of the normal brain (Fig. 3*A*) would make it easier to detect the CT decrease in the right hemisphere. Taking these findings together, we conclude that the mass effect of a glioma, and/or an associated tumor-infiltrated edema, can extend to almost all cortical regions that are detectable by the SBM analyses.

We also observed cortical structural changes in the peripheral regions of a glioma, which were different from those in the contralateral hemisphere. The patient group showed a decreased FD in the left frontal cortex (Fig. 4*C*), resulting in a significantly lower FD compared with the right homolog (Fig. 5*B*). Interestingly, the FD > CT difference for the patient group decreased in the left premotor cortex (Fig. 6*C,D*), within the peripheral region of the lesion overlap (see Fig. 1*A*), while CT in the left frontal cortex itself was well preserved (Fig. 2*C*). Diffuse gliomas are characterized by an extensive, diffuse infiltration of tumor cells in the neuropil, that is, the dense network of interwoven neuronal and glial cell processes (Claes et al. 2007). The infiltrative nature of a glioma would thus be compatible with the preserved CT and decreased FD. In an animal study, laminin deposits have been observed in the border zone between the normal region and implanted glioma cells (Pedersen et al. 1993). Infiltrative glioma growth has also been reported in the peripheral regions of a glioma with DTI and MR spectroscopic imaging (Wright et al. 2009). It is possible that the structural changes due to tumor infiltration and tumor-infiltrated edema contribute to the decrease in FD with the relative preservation in CT.

Another possible explanation for the cortical structural changes might be cerebral plasticity caused by the presence of a glioma, similar to the cerebral reorganization after a brain injury (Desmurget et al. 2007). This possibility would explain why most patients with a glioma appear either normal or only slightly impaired under standard neurological assessments (Duffau 2005). Preoperative neuroimaging studies have shown that tumor invasions trigger reorganizations not only in the ipsilateral hemisphere of a glioma, but also in the contralateral hemisphere (Ojemann et al. 1996; Meyer et al. 2003; Kinno et al. 2014). Cortical reorganization within injured and perilesional structures should be regarded as primary changes, while distant regions in the ipsi- and contra-lesion hemispheres would be further recruited when the first changes become insufficient (Desmurget et al. 2007). In association with these functional changes, cortical structures would be globally affected; a recent VBM study reported an increased GM volume in the insular region contralateral to an insular glioma (Almairac et al. 2018). However, we observed neither increased CT nor increased FD for the patient group, when compared with the control group, which would limit the possibility of cortical thickening. The underlying mechanisms could include changes in cell size, spine density, neural/glial cell genesis, and even changes in blood flow or interstitial fluid (May et al. 2007; Čeko et al. 2013), as well as WM enlargement leading to cortical thinning. The WM enlargement is often associated with developmental disorders (Herbert et al. 2004), but WM enlargement could be just a physiological side effect of elevated activity, as oligodendrocytes are responsive to neuronal activity (Barres and Raff 1999).

Some methodological issues should also be discussed. First, it has been reported that microscopical tumor cells are observed *within* the glioma visualized by the T2-weighted MR image but are less commonly found outside the glioma (Harpold et al. 2007). Moreover, astrocytic and oligodendroglial gliomas can be detected by complete or near-complete hyperintense signals on a T2-weighted MR image, together with hyperintense signals on a fluid attenuation inversion recovery image (Weller et al. 2017). To identify abnormal regions due to glioma to the greatest extent possible, we included all T2-hyperintense regions in the tumor volume. It is possible that such T2-hyperintense regions also included brain edema, although the contribution of edema was limited for our cases, as shown in Figure 1*B–D*. Secondly, to correctly estimate CT and FD in the SBM analyses, the accuracy of segmentation and normalization is essential. It has been reported that the DARTEL algorithm is suitable for accurate normalization of the brain with a lesion (Ripollés et al. 2012), and previous VBM studies for patients with a glioma successfully achieved the segmentation and normalization with this algorithm (Almairac et al. 2018; Zhang et al. 2018; Yuan et al. 2020). In the present study, we used DARTEL and visually inspected all resultant images for the absence of artifacts or deformation, which passed the quality control and homogeneity control algorithms implemented in the CAT12 Toolbox. In addition, the SBM results in the right hemisphere were free from potential methodological issues, because no visible midline shift was present on the normalized T1-weighted MR images. Further methodological progress would make the SBM analyses more accessible for examining patients with focal lesions.

## Conclusion

The results of our SBM analyses demonstrate the global mass effects and infiltrative nature of a glioma on the cortical structure in both hemispheres. Such detailed assessment is particularly useful, because the infiltrative nature of a diffuse glioma often makes it difficult to define the boundaries of the tumor. Indeed, conventional radiological examinations with computed tomography and standard MRI tend to significantly underestimate the extent of diffuse infiltrative glioma growth (Claes et al. 2007), and infiltrative glioma cells have been histologically detected beyond the hyperintensive area on T2-weighted images (Ganslandt et al. 2005). The importance of studying patients with a glioma continues to increase not only within the clinical field (Taphoorn and Klein 2004) but in basic neuroscience as well (Kong et al. 2016). Comparative analyses of CT and FD data based on high-resolution 3D-MRI could become a valuable tool for the examination of all brain regions affected by a glioma.

## Notes

We thank N. Komoro for the technical assistance and H. Matsuda for the administrative assistance. *Conflict of Interest*: None declared.

## Funding

Grant-in-Aid for Scientific Research (C) (#JP17K01978) from the Japan Society for the Promotion of Science; grant from the Brain Science Foundation.
